# News and Notes

**Published:** 1899-11

**Authors:** 


### *NEWS AND NOTES.*

DR. W. J. SHAW and Miss Daisy Cheney were united in marriage at Rome, Ga., October 25th. Congratulations.

DR. J. R. TUCKER, of Chipley, Ga., died in that place on October 5th, at the age of 28. He was buried with Masonic honors.

DR. W. A. CULBERSON, of Cave Spring, Ga., died in that city on October 19th, after a short illness. He was well-known in his section of the State.

THE *Tri-State Medical Journal* has again changed its name. It is now called the *Interstate Medical Journal;* the editorial staff and business management remaining the same. The change is certainly a commendable one.

DR. J. C. RIPPARD has been appointed Chief Surgeon to the Plant Railway System in place of Dr. F. H. Caldwell, who resigned. Dr. Rippard is a resident of Waycross, Ga., where is located the hospital.

THE *American Journal of Surgery and Gynecology* is a "hustling journal," although frequently containing antique articles. We fear its distinguished editor is not a close proof-reader, judging by the numerous typographical errors.

A COMBINATION of the *Atlanta Medical and Surgical Journal* with the *Southern Medical Record* has been made—much to the credit of their managers. THE JOURNAL-RECORD is now one of the best publications in the South.—*American Journal of Surgery and Gynecology.*

ACCORDING to the *Railway Surgeon* for August 22d, Judge Colt, of the U. S. Circuit Court, has decided that surgical instruments imported to this country are not dutiable, since they all come under the head of "scientific instruments."

---

IT is reported that the Board of Health of California is proposing to establish a quarantine against all persons afflicted with tuberculosis and the isolation of such as already have the disease. This, we think, is a very unnecessary and unwise legislation.

---

THE Hospital of St. Vincent, in Norfolk, Va., was recently destroyed by fire almost *in toto*. It was, perhaps, the most elegant and most thoroughly equipped hospital in the South, and reports say that the insurance upon it will not nearly cover the loss.

---

DR. WILLIAM H. HOWELL, Professor of Physiology at the Johns Hopkins University, has been made dean of the medical school. He is not superintendent of the Johns Hopkins Hospital, as has been reported, but Dr. Henry M. Hurd continues to hold that position.

---

IT is reported that the milk sold in large cities is now being preserved by small amounts of formaldehyd. The president of the Indianapolis Board of Health has shown conclusively that even small quantities of formaldehyd interfere with digestion, and therefore all milk should be examined.

---

"Do you make much out of your apples?" asked the visitor. "Oh, pretty considerable," answered the farmer, "but I've got a son up in the town who makes more out of the apples in a month than I make the whole season." "A farmer, is he?" "No; he's a doctor. I'm talking about green apples now."—*The Statesman, Yonkers.*

IT will be a year October 23 since young Dr. Mueller lost his life in the "laboratory plague epidemic in Vienna." An imposing memorial to him will be unveiled on that date, in the grounds of the General Hospital opposite Nothnagel's clinic, the expenses defrayed by subscriptions from the profession.

---

PROF. BUNSEN, of Heidelberg, the great chemist and physicist, recently died at the good age of 88. His contributions to the science of chemistry were as great as those of any man that ever lived. The Bunsen burner is probably better known to chemical students than any other of his original inventions.

---

**NOTICE.—This JOURNAL and THE INTERNATIONAL JOURNAL OF SURGERY one year for $1.00 cash, to new subscribers. No out-of-town checks accepted unless 10c. is added for cost of cashing.**

---

THE University of Chicago has a new and unique branch in the Chicago Physiological School for the training of nervous and backward children. It is said to be the first of its kind in the world, and is intended as a home for boys and girls who are unable to cope with normal children owning to illness or infirmity.

---

THE next meeting of the Mississippi Valley Medical Association will be held in Asheville, N. C. The new officers are: President, Dr. H. D. Moyer, Chicago; Vice-Presidents, Drs. H. A. Cordier, Kansas City, and S. R. Collings, Hot Springs; Secretary, Dr. H. E. Tuley, Louisville; Treasurer, Dr. D. S. Reynolds, Louisville.

---

***NOTICE.—This JOURNAL and THE INTERNATIONAL JOURNAL OF SURGERY one year for $1.00 cash, to new subscribers. No out-of-town checks accepted unless 10c. is added for cost of cashing.***

MRS. NABOR: "And so the doctor ordered you to give your husband whisky for his rheumatism. Does it seem to do him any good?"

MRS. NEXDOOR: "John says it does him lots of good, but I notice the pains come upon him more frequently than ever."—*Ohio State Journal.*

---

**NOTICE.—This JOURNAL and THE INTERNATIONAL JOURNAL OF SURGERY one year for $1.00 cash, to new subscribers. No out-of-town checks accepted unless 10c. is added for cost of cashing.**

---

THE sum of $50,000 has recently been given to the University of Pennsylvania, the object of which will be toward defraying part of the cost of the new dormitories now in the course of construction. The name of the donor has been withheld for the present. The property at Thirty-fourth and Locusts streets has been acquired by the trustees, and on this site the new laboratory of medicine and the new gymnasium will be built.

---

***NOTICE.—This JOURNAL and THE INTERNATIONAL JOURNAL OF SURGERY one year for $1.00 cash, to new subscribers. No out-of-town checks accepted unless 10c. is added for cost of cashing.***

---

A SPECIAL committee of the Illinois State Board of Health, appointed to investigate the advisability of establishing a State institution for the care of consumptives, has made its report to that body and urges that an appropriation of $200,000 be granted by the legislature for the establishment of a sanatorium. Action has been deferred until the next meeting of the Board. It is stated that the project has the approval of the Governor.

